# Modern advances in spinal cord regeneration: hydrogel combined with neural stem cells

**DOI:** 10.3389/fphar.2024.1419797

**Published:** 2024-06-27

**Authors:** Oksana Rybachuk, Yuliia Nesterenko, Valeriia Zhovannyk

**Affiliations:** ^1^ Bogomoletz Institute of Physiology NAS of Ukraine, Kyiv, Ukraine; ^2^ Institute of Genetic and Regenerative Medicine, M. D. Strazhesko National Scientific Center of Cardiology, Clinical and Regenerative Medicine, National Academy of Medical Sciences of Ukraine, Kyiv, Ukraine

**Keywords:** neural stem/progenitor cells (NSPCs), hydrogel, neuron, glia, differentiation, scar, implantation, spinal cord injury/s (SCI)

## Abstract

Severe spinal cord injuries (SCI) lead to loss of functional activity of the body below the injury site, affect a person’s ability to self-care and have a direct impact on performance. Due to the structural features and functional role of the spinal cord in the body, the consequences of SCI cannot be completely overcome at the expense of endogenous regenerative potential and, developing over time, lead to severe complications years after injury. Thus, the primary task of this type of injury treatment is to create artificial conditions for the regenerative growth of damaged nerve fibers through the area of the SCI. Solving this problem is possible using tissue neuroengineering involving the technology of replacing the natural tissue environment with synthetic matrices (for example, hydrogels) in combination with stem cells, in particular, neural/progenitor stem cells (NSPCs). This approach can provide maximum stimulation and support for the regenerative growth of axons of damaged neurons and their myelination. In this review, we consider the currently available options for improving the condition after SCI (use of NSC transplantation or/and replacement of the damaged area of the SCI with a matrix, specifically a hydrogel). We emphasise the expediency and effectiveness of the hydrogel matrix + NSCs complex system used for the reconstruction of spinal cord tissue after injury. Since such a complex approach (a combination of tissue engineering and cell therapy), in our opinion, allows not only to creation of conditions for supporting endogenous regeneration or mechanical reconstruction of the spinal cord, but also to strengthen endogenous regeneration, prevent the spread of the inflammatory process, and promote the restoration of lost reflex, motor and sensory functions of the injured area of spinal cord.

## 1 Introduction

Spinal cord injuries (SCI) of various genesis lead to the loss of axons (and neurons), which in turn leads to impaired sensory and motor functions, disability and often death. In 2019, there were 20.6 million people worldwide living with spinal cord injury (Safdarian et al., 2023). All traumatic injuries of nervous tissue lead not only to physical limitations, reduced quality of life, and social losses, but also to significant financial losses at the state level (up to 56 billion dollars in the USA) (Ai et al., 2013; Tian et al., 2015). That is why an important issue for regenerative biology is the investigation of nervous tissue regenerative potential, the ways of its activation, increase, as well as the search for alternative methods of effective structural and functional restoration of injured tissue.

Injury of spinal cord (SC) tissue leads to activation of internal regenerative potential, which is extremely limited by activation of reactive astrogliosis with subsequent glial scar formation at the injury site (Okada et al., 2018; Li et al., 2020; Yu et al., 2021). Such a pathological process prevents the regeneration of axons and functional restoration of SC tissue. In addition, tissue injury leads to the appearance of such proteoglycans as chondroitin and keratin sulfate in the extracellular matrix. These inhibitory molecules of the extracellular matrix are synthesized by reactive astrocytes of glial scar and make it extremely difficult to repair damaged tissue (Ai et al., 2013; Yuan and He, 2013; Takazawa et al., 2018).

A lot of studies in regenerative biomedicine are dedicated to elucidating neural stem cell (NSC) properties for their further use in nerve tissue regeneration after SCI (Son et al., 2023; Xu et al., 2023). The main and important characteristics of NSCs are their ability for self-renewing and differentiation into nerve tissue cells (Vieira et al., 2018; Zhu et al., 2021; Yale et al., 2023). Moreover, NSCs synthesize cytokines, growth factors and other trophic factors that provide a neuroprotective influence on neighbouring cells, thereby affecting their survival (De Gioia et al., 2020; Dause et al., 2022). In addition to NSCs, liver stem cells, neural crest derivatives (isolated from skin or hair follicles), bone marrow stem cells, mesenchymal stem cells (MSCs), and Schwann cells are used to restore damaged or lost parts of nervous tissue (Hui et al., 2011; Stoltz et al., 2015). However, the use of stem cells is very often ineffective due to the presence of a glial scar at the injury site and the toxicity of the recipient’s extracellular environment (Nicaise et al., 2022; Han et al., 2023).

Since the 1990s, one of the progressive directions of regenerative biology has been tissue engineering, which proposes the use of polymer matrices of various origins for damaged/lost tissue regeneration, in particular nerve tissue, because of diseases, ageing or trauma (Han et al., 2020; Akhtar et al., 2023; Zarrintaj et al., 2023). The main goal of tissue engineering is the development and synthesis of polymer matrices with mechanical, chemical and physical characteristics similar to those of native tissue. After all, the ideal matrix should promote differentiation, cell growth into a polymer frame, integrate into the recipient`s tissue, without causing an inflammatory response, and mimic the extracellular matrix (ECM), namely, provide trophic support and, similar to the native, biochemical composition (Snigdha and Sabu Thomas, 2017; Radulescu et al., 2022; Revete et al., 2022; Sánchez-Cid et al., 2022). In addition, polymer matrixes must completely degrade or transform into non-toxic metabolites within the living organism tissue (Revete et al., 2022; Sánchez-Cid et al., 2022).

Nowadays, there is a significant number of biological polymer matrixes that differ in origin, method of synthesis, physical structure, chemical composition, and type of cross-linking agent, and are used as implants for spinal cord tissue and nerve processes restoration (Ai et al., 2013; Maitz, 2015; Stratton et al., 2016; Ganz et al., 2017). To restore the structure of injured SC, implants based on natural polymer materials are often used—collagen, laminin, fibronectin, hyaluronic acid, cellulose, methylcellulose, agarose, chitosan, etc. (Ahmadi et al., 2015; Yeh, 2018; James et al., 2019). Synthetic matrices based on polyolefin, silicones (polydimethylsiloxane (PDMS)), methacrylates (polymethylmethacrylate (PMMA), PHEMA, PLGA, PGA, PLLA)), polyesters (polycarbonates (PC)), polyethylene terephthalate (PET, dacron), polyethersulfone (PES), polyethers, polyurethanes (polytetrafluoroethylene (PTFE), PVA, PVC)) etc. have also been actively used for damaged nerve tissue regeneration (Ahmadi et al., 2015; Snigdha and Sabu Thomas, 2017; Yeh, 2018; Ligorio and Mata, 2023).

In recent years, a large number of studies have been devoted to elucidating effectiveness of hydrogel matrices during implantation in the SCI area (Lv et al., 2022; Radulescu et al., 2022; Qin et al., 2023; Sun et al., 2023). It was shown that hydrogels, as synthetic and semi-synthetic matrices, provide appropriate conditions for cell adhesion and cellular remodelling in a three-dimensional structure (González-Díaz and Varghese, 2016; Akther et al., 2020; Ligorio and Mata, 2023). In addition, hydrogel scaffolds can retain proteins, growth factors, and components necessary for cell growth and differentiation (Hejčl et al., 2013; Khaing et al., 2014; Ahmadi et al., 2015; Assunção-Silva et al., 2015; James et al., 2019). Hydrogels’ physicochemical characteristics (flexibility, elasticity, and density—properties that affect the mechanics of the hydrogel implant, thereby preventing additional compression on the tissue of the recipient) make it possible to successfully combine them with each other, improving structure and functionality; also combine them with stem cells (populate), thereby increasing nerve tissue regeneration efficiency (Khayambashi et al., 2021; Li et al., 2022; Rybachuk et al., 2023b; Hasanzadeh et al., 2023; Giorgi et al., 2024; Politrón-Zepeda et al., 2024). For instance, a biocompatible macroporous hydrogel based on poly-N-hydroxypropylmethacryamide (pHPMA) in recipient tissue can mimic the natural environment for cells, in particular, NSCs, that will further differentiate into neurons, astrocytes, and oligodendrocytes; can encapsulate not only stem cells but also bioactive molecules, which makes it possible to further regulation of introduced cells response, activation and increase the regenerative potential of tissue at the injury site (Trimaille et al., 2016).

The number of *in vitro* and *in vivo* studies on the population of hydrogels with stem cells, in particular, NSCs, is still insignificant, but it is already possible to confidently indicate the effectiveness of this approach for SC tissue regeneration after injury. In this review, we will consider data that confirm the effectiveness and feasibility of using hydrogels populated with stem cells in SCI. First, we consider stem cell transplantation as one of the options for improving the condition after the SCI; we indicate the positive and negative sides of this method. We also analyze information about currently available matrix materials (primarily hydrogels), which should be regarded as the most promising for neuroengineering applications; we identify the main characteristics/criteria of hydrogels that should be considered when selecting a material for stem cell population and subsequent implantation.

## 2 NSC transplantation in SCI

SCI of various provenance causes the loss of a large number of nerve processes, including neurons, resulting in a considerable decrease in sensitivity and decreased movement of the body part below the site of injury. Unlike other vertebrates, the regenerative potential of the mammalian CNS is quite limited, despite its inherent so-called spontaneous recovery, i.e., the set in endogenous regenerative processes in the affected area (Steeves, 2015; Khorasanizadeh et al., 2019; Tran et al., 2022). Insignificant ability to recover caused by suppression of all reparative processes, the appearance of myelin debris, the absence of a sufficient amount of intercellular substance and violation of the natural ratio of its component molecules as a result of tissue injury. Precisely intercellular substance, that consists of a linked macromolecules network, which forms a highly hydrated gel-like structure that serves not only as a physical support for cells but also ensures the functional integrity of the organ. Due to the intercellular substance, both the distribution of some signalling molecules and factors necessary for normal tissue functioning and regeneration in case of damage are also controlled (Casarosa et al., 2014; Marquardt and Heilshorn, 2017).

In addition, during SC tissue injury, in most cases, neurons are replaced by astrocytes, thereby creating a glial scar (Okada et al., 2018; Yu et al., 2021). Even though in this way the injury site is isolated from the neurons, the formation of glial scar also prevents normal electrical conductivity between neurons, which in turn affects all nerve tissue functional state (Casarosa et al., 2014). That is why the up-to-date task of common regenerative biology is the search for ways to restore tissue, in particular SC tissue, after traumatic damages of various genesis.

Today, replacement repair surgery is one of the developed procedures for reconstructing injured tissue (replacement of lost cells) (Yuan, 2009). Thus, to strengthen the endogenous regeneration of damaged SC parts and counteract the development of inflammatory processes, the replacement of nervous tissue damaged areas with stem cells, in particular NSCs, is carried out (Li et al., 2021; Xue et al., 2021; Guo et al., 2022). As known, NSCs secrete a large number of active compounds by an autocrine/paracrine mechanism. These active compounds (NSC secretomes) have an important therapeutic role. The NSC secretome promotes the regeneration of nervous tissue, particularly SC: improving the survival of recipient nerve cells, neuroplasticity and neuroimmune modulation (Dause et al., 2022). The autocrine mechanism consists of synthesising cytokines, growth factors and extracellular matrix molecules by stem cells for their self-maintenance, cell migration and adhesion (Dause et al., 2024). Autocrine regulation is ensured by activation of the expression of sonic hedgehog (SHH), platelet derived growth factor (PDGF), chondroitin sulfate proteoglycans (CSPGs), apolipoprotein E (ApoE) and pituitary adenylate cyclase-activating peptide (PACAP), vascular endothelial growth factor (VEGF), insulin-like growth factor 2 (IGF2), milk fat globule-EGF 8 (MFGE8), Wingless and Int-1 (WNT), glycosylated cystatin C (CCg) and stem cell-derived neural stem/progenitor cell supporting factor (SDNSF). Also, NSCs secrete aggrecans, neurocans, and phosphocans, which increase the survival of NSCs in the recipient’s tissue (Gong et al., 2020; Dause et al., 2022; 2024). Moreover, through a paracrine mechanism, NSCs can regulate the microenvironment under pathological conditions (Wang et al., 2019; Zhang et al., 2020; Pajer et al., 2021); due to the paracrine mechanism, NSCs can perform intercellular responses. NSC secretome includes chemokines, cytokines, early inflammatory cytokines, growth factors, regulatory factors of stem cells, extracellular vesicles, etc. It is these molecules that are important in the regulation of critical biological processes such as cell survival/viability, proliferation and differentiation, immune regulation, anti-apoptosis and stimulation of neighbouring cells in tissues (Skalnikova et al., 2011; Kirby et al., 2015; Overall et al., 2016; Hu et al., 2018; Wang et al., 2019; Zhang et al., 2020; Pajer et al., 2021; Gilbert et al., 2022).

It has been proven that NSCs differentiate into neurons when transplanted into injured SC (Kong et al., 2021; Mahadewa et al., 2021; Xue et al., 2021); activation of axon growth occurs—sprouting of axons by 10 mm into the white matter of the recipient`s SC (Pereira et al., 2019). In addition, transplanted NSCs differentiate not only into neurons, which contribute to the active synapse formation with neurons of the recipient’s SC but also into glial cells (Dulin and Lu, 2014; Zhao et al., 2017; Guo et al., 2022).

Due to NSCs can differentiate into neurons and glial cells, they are used for the regeneration of the recipient’s axons and the restoration of the SC conducting pathways. In turn, axons in the recipient`s SC are capable of forming corticospinal, serotonergic, noradrenergic, propriospinal and sensory axons with transplanted NSCs (Hou et al., 2020; Zawadzka et al., 2021). Beyond that, transplanted NSCs can reduce secondary damage to nerve tissue. However, to activate the internal regenerative processes of axons in injured SC, it is necessary to use additional therapeutic approaches, in particular, neurotrophin introduction. Genetic approaches can also be used, namely, deletion of phosphatase and tensin homolog (PTEN) and overexpression of Krüppel-like factor 7 (Dulin and Lu, 2014).

Transplanted NSCs survive even in conditions of chronic SCI, contributing to damaged axon recovery and reducing the cavity in the nerve tissue. And NSCs differentiated into oligodendrocytes promote axon remyelination while NSCs differentiated into astrocytes provide vascularization of nervous tissue and facilitate growth/neuroprotective factors synthesis; differentiated motoneurons and interneurons form new neuronal connections between recipient nerve cells and differentiated NSCs (Pereira et al., 2019; Li et al., 2021; Suzuki et al., 2022).

On the other hand, it is known that NSCs play an important role in scar formation during the first 2 weeks after SCI. Active replacement of lost neurons occurs due to the differentiation of endogenous NSCs into astrocytes, which form the scar, and into oligodendrocytes progenitors, which later partially provide myelination of damaged axons (Nicaise et al., 2022). In such a way, the damage site is isolated from intact neurons, but the formation of a glial scar opposes the normal electrical conduction between neurons, which in turn affects the functional state of the entire nervous tissue (Ai et al., 2013; Yuan and He, 2013; Casarosa et al., 2014; Takazawa et al., 2018).

Nevertheless, transplantation of exogenous NSCs is considered an alternative approach to the regeneration of injured SC tissue. After all, there is an activation of neurogenesis and a decrease in inflammation, which occurs as a result of scar formation. This way of nerve tissue regeneration is based on the secretion of NSC growth factors, which create a supportive extracellular milieu in a paracrine way. The extracellular environment prevents further development of neurodegenerative processes due to scar formation and stimulates regenerative processes in damaged nerve tissue (Nicaise et al., 2022).

However, clinical findings suggest that this regenerative approach results in only a short-term improvement in symptoms, while injury degree persists at a higher level than expected. The low level of NSC survival in damaged areas (5%–20%) is caused by the presence of transplanted cells in a non-vascularized injured area with haemorrhages (Park et al., 2012; van Gorp et al., 2013; Oliveri et al., 2014; Zhang and He, 2014; Powell and Davidson, 2015; Assinck et al., 2017). Moreover, the post-traumatic microenvironment very often turns out to be extremely toxic for stem cells due to myelin debris appearance, glial scar formation, and release of molecules by damaged tissue that inhibit NSCs growth and differentiation (Nicaise et al., 2022; Han et al., 2023). Another problem with this approach is the need for a large number of cells for transplantation. Thus, although conventional cell transplantation is promising in restoring small damaged areas of grey matter, this method remains inefficient in SCI (Khazaei et al., 2017).

## 3 Hydrogels and spinal cord repair

As studies of the last decade have shown, the implantation of polymer matrices is also a promising direction for the restorative treatment of SCI (Cheng et al., 2022).

Nowadays, there are a lot of biological polymer matrixes that differ in origin, method of synthesis, physical structure, chemical composition, and type of cross-linking agent, and are used in the form of implants for the brain and spinal cord nerve tissue, nerve processes restoring (Ai et al., 2013; Maitz, 2015; Stratton et al., 2016; Ganz et al., 2017). Recently, hydrogels are most often used, both for direct implantation and as a material for combined implant variants manufacture.

Hydrogels are polymers with a hydrophilic structure that allows them to absorb a lot of water and other biological fluids. Depending on the polymer (polymers) properties, as well as on the nature and density of bonds, such structures may contain different amounts of water in equilibrium; usually in a hydrated (swollen) state, the mass fraction of water in hydrogel significantly exceeds the mass fraction of the polymer (Ahmed, 2015). Hydrogels are known to absorb 99% w/w of their dry weight without dissolution (Ebhodaghe, 2022). The high ability of hydrogels to absorb water ensures their ability to facilitate nutrient diffusion (Radulescu et al., 2022).

Hydrogels can possess such biomimetic qualities as flexibility, softness, excellent absorbency, non-toxicity, biocompatibility, biodegradability and adjustable mechanical properties (Sánchez-Cid et al., 2022). In addition, their considerable flexibility and elasticity, similar to the natural ECM, provide necessary biochemical and structural support for surrounding cells and influence tissue formation (Radulescu et al., 2022).

The advantages of hydrogels as matrices lie in their ability to create a trimeric framework for neuronal regeneration and axon elongation, which initiates cell growth, proliferation, and cell migration. Also, hydrogels contribute to the delivery of SC precisely to the damaged site, reducing cell loss in the adjacent tissue. In addition, hydrogels slowly release medicinal substances or bioactive factors introduced into them, which contributes to a more stable and long-term recovery of SC tissue (Perale et al., 2011).

The use of hydrogels, namely, collagen, gelatin, fibrin, chitosan, alginate, laminin, agarose, dextran, and hyaluronic acid-based hydrogels are considered effective because collagen is the main component of the extracellular matrix in CNS. *In vitro* experiments show that collagen favours neural cell proliferation and differentiation and prevents the development of neurocollagen gliosis. Collagen hydrogels promote nerve cell migration, axon growth and regeneration, brain-derived neurotrophic factor (BDNF) and neurotrophin-3 (NT-3) synthesis (Lv et al., 2022).

Alginate hydrogels are used to deliver drugs or cytokines to the injury site. The effectiveness of hydrogels based on hyaluronic acid is related to the fact that hyaluronic acid is also a natural component of the SC extracellular environment: therefore, the presence of such a component reduces inflammation and reduces fibrous scar size after SCI (Lv et al., 2022).

Chitosan polymers are also actively used for nervous tissue regeneration in the form of stents, which help to reduce inflammation and fibrous scar formation, as well as to restore SC vessels and axons. And fibronectin hydrogels actively adhere to the recipient`s tissue and have a neuroprotective effect by regulating ischemia-induced necrosis after SCI (Wang et al., 2022; Lv et al., 2022).

Some studies also confirm the effectiveness of using hydrogels based on biodegradable oligopolyethylene-glycol-fumarate for damaged SС tissue regeneration (Chen et al., 2011). Moreover, scientists have recently used biodegradable polymers to regenerate individual nerve endings. For instance, many experimental works are devoted to the investigation of implanted PLGA structure’s impact using fibrin glue in the injured area of SC. Research results indicate that such polymer implants contribute to the restoration of axons in peripheral nerve endings (Grahn et al., 2014). And natural polysaccharide alginate is used as a hydrogel to imitate the ECM of nervous tissue (Lv et al., 2022).

During the last 2 decades, natural hydrogels have been gradually replaced by synthetic hydrogels, which have a long service life, high water absorption capacity, and high gel strength. After all, synthetic polymers usually have well-defined structures that can be modified to provide individual degradability and functionality (Ahmed, 2015). Most hydrogels are completely artificial or petrochemical in origin and are made from acrylic monomers. In the industrial production of hydrogel, acrylamide (AM), acrylic acid (AC) and its sodium or potassium salts are most often used (Ahmed, 2015).

Due to their porosity, hydrogel matrices, in particular synthetic ones, promote cell migration and blood vessel growth. In comparison with hydrogels of natural origin, it is possible to independently determine the physical parameters of synthetic hydrogels, their size and the number of pores. In addition, synthetic hydrogels do not cause an immune response (Cai et al., 2023).

Today, some clinical applications of hydrogels are known, in particular, for wound healing, bone regeneration, framework formation to provide a biomimetic 3D microenvironment of nervous tissue, drug delivery, intraocular lenses, contact lenses, corneal prostheses, orthopaedics, biosensors, hemostatic bandages, etc. (Revete et al., 2022). Hydrogel scaffolds can retain proteins, growth factors and components necessary for cell growth and differentiation (Hejčl et al., 2013; Khaing et al., 2014; Ahmadi et al., 2015; Assunção-Silva et al., 2015; James et al., 2019). In most cases, hydrogels are used as homogeneous soft materials with homogeneous volumetric properties. To better mimic the anisotropic and complex structures of body tissues, hierarchical hydrogels containing multiple layers with different biochemical signals and mechanical properties play a crucial role in biomedical applications (Revete et al., 2022).

Constructions based on a mixture of synthetic biodegradable polymers PLGA, PLC, and poly-2-hydroxyethylmethacrylate (pHEMA) are also used to restore SC tissue, PLA macroscopic sponges are also effective for restoring SC axonal pathways (Straley et al., 2010; Shrestha et al., 2014; Gao et al., 2016).

A significant amount of investigations are devoted to the examination of biocompatible macroporous hydrogel based on poly-N-hydroxypropylmethacrylamide (pHPMA) properties (Pertici et al., 2013). pHPMA-hydrogel and its derivatives are synthetic polymers with high biocompatibility. One of the derivatives of pHPMA is NeuroGel, the three-dimensional structure of which is represented by a contacting network of macromolecules of hydrophilic monomers that swell in an aqueous environment. The internal structure of pHPMA-hydrogel (Neurogel) is represented by a set of pores of different diameters (2–300 nm), which allow micro-, macromolecules, and cells, including during the process of vascularization, to move freely within the matrix (Woerly et al., 1999; 2001; Pertici et al., 2013). It is also known that NeuroGel prevents glial scar formation in injured tissue; at the same time, free tissue (spared tissue) in the centre of injury can improve motor functions due to the reorganization of nerve pathways below the injury site (Woerly et al., 2004; Ma et al., 2022; Ma et al., 2022). Such a hydrogel matrix in a recipient`s tissue can mimic natural environmental conditions for stem cells, in particular NSCs, which will be able to differentiate freely into neurons, astrocytes, and oligodendrocytes in the future. In addition, pHPMA-hydrogel derivatives can encapsulate not only stem cells but also bioactive molecules, which makes it possible for further regulation of introduced/populated cell response, and activation and increase regenerative potential of tissue at the injury site (Trimaille et al., 2015).

As known, disruption of microenvironment composition as a result of SCI often leads to nervous tissue infection. Hydrogels with anti-inflammatory and antibacterial properties contribute to the reduction or complete elimination of infection processes, which significantly increases the efficiency of tissue regeneration (Sun et al., 2023).

In recent years, conductive hydrogels have gained popularity—a class of organic materials with unique electrical and optical properties similar to those of inorganic semiconductors and metals (Tomczykowa and Plonska-Brzezinska, 2019; Xuan et al., 2023). Due to high electrical activity, such matrices promote cell adhesion, their growth and proliferation. Polypyrrole (PPy), polyaniline (PANi), polythiophene (PT), its derivatives, and poly (3,4-ethylenedioxythiophene) (PEDOT) are most often used for SC tissue regeneration (Qin et al., 2023).

Peptide hydrogels are also currently being actively used for wound healing, drug delivery, and in combination with stem cells in SCI (Sun et al., 2023). Peptide hydrogels based on insulin-like growth factor 1 (IGF1c) contribute to neuronal activation, proliferation and differentiation of populated NSCs for further implantation in injured SC. In addition, this type of matrix increases the survival of NSCs due to the reduction of acute inflammation in the early stages of the post-traumatic period and also enhances the growth of axons and suppresses the development of reactive astrogliosis. Such properties of the hydrogel contribute to the improvement of motor functions in animals with SCI (Wang et al., 2023).

It is known that the self-assembling peptide (SAP) group of highly organized hydrogels has biomimetic tunnelling (Mondal et al., 2020; Gelain et al., 2021). SAP hydrogels can retain water, have high biocompatibility and are similar to the extracellular matrix in many ways. The advantages of such synthetic hydrogels are their characteristics, such as porosity, elasticity, and drug delivery rate. SAP is characterized by a slow and sustained release of active cytokines, and the release kinetics depend on the SAP sequences and their type (Mondal et al., 2020). Injections of SAP hydrogels loaded with ChABC (Chondroitinase ABC) have been shown to promote neuronal regeneration and recovery of functional activity in rats with chronic SCI. In addition, it was demonstrated that under *in vitro* conditions, ChABC enzymatic activity (usually lasting less than 72 h) released from SAP was detected in significant amounts up to 42 days (Raspa et al., 2021).

Moreover, RGD and BMHP motifs contained in SAP contribute to the proliferation and differentiation of introduced NSCs. KLPGWSG and FAQRVPP motifs activate NSC differentiation into neurons and oligodendrocytes. The inclusion of 3 motifs (LDLK) in such hydrogels promotes the differentiation of NSCs into neurons, astrocytes, and oligodendrocytes (Gelain et al., 2021).

In general, the inclusion of RGD and PDGF-A protein motifs in the composition of hydrogels also improves their characteristics and, accordingly, the regeneration of nerve tissue. Thus, the inclusion of such peptides in hydrogels based on hyaluronic acid, collagen, fibrin, alginate, chitosan, gelatin, agarose, and methylcellulose promotes early survival, integration, migration, and differentiation of introduced stem cells into the matrix. Modification of the hydrogels with such peptides results in enhanced axon growth and electrical conductivity, thus replacing the cavity formed as a result of the injury (Wang et al., 2022; Chen et al., 2024).

### 3.1 Necessary characteristics of hydrogels for their use in neuroengineering

As is well known, hydrogels in regenerative medicine are used as scaffolds to provide structural integrity and volume for cellular organization and morphological orientation, serve as tissue and bioadhesive barriers, act as reservoirs for drugs, deliver biologically active agents that promote natural recovery process, encapsulate and deliver cells. Due to their high water content and physical and chemical properties, hydrogels are suitable for use even in soft organs such as the brain and spinal cord (Revete et al., 2022; Sánchez-Cid et al., 2022).

The elasticity of hydrogels varies within 3–300 kPa, which corresponds to the elastic properties of SC tissue. Within the hydrogels, the recipient`s SC neurons are characterized by a longer axon length, and also provide an appropriate composition of extracellular environment for nerve tissue electrical stimulation to accelerate axon regeneration. This ability is based on hydrogels’ ability to inhibit reactive oxygen species synthesis in the area of injured SC neurons and, thus, to prevent apoptosis of intact SC cells and promote regeneration of already injured SC neurons. Hydrogels are also effective for filling of cavities formed in damaged nerve tissue: they create channels for the passage of electrical signals in SC (Sun et al., 2023).

Therefore, hydrogels as materials for further use in (neuro) tissue reconstruction should have the following characteristics/criteria:

*Biocompatibility*: the goal of biocompatibility of any hydrogel evaluation is a limitation of toxic effects that can be caused in the body. The three main factors to consider during evaluation are the healing process, inflammation, and immune response known as immunotoxicity (Lee and Mooney, 2001; Griffith, 2002; Drury and Mooney, 2003; Brandl et al., 2007; Chamkouri, 2021; Radulescu et al., 2022). By applying stimuli such as light, pH, temperature or magnetic field, hydrogels can release various active substances that have been “sewn” into their structure, which can be one of the options for local effects on various pathologies (Radulescu et al., 2022).

*Adhesion*: hydrogel matrices are known as inherently hydrated materials, and their adhesion depends on sparse and loose adhesive junctions that are surrounded by water (Yang. et al., 2022).

*Porosity*: one of the most important properties of hydrogels is porosity. This property is important for biomaterial structure, which ensures oxygen and nutrients supply to cells and the removal of cellular debris. In addition, the porous structure of the scaffold also affects cell migration and tissue integration. The pore size is crucial for the depth of cell penetration into the hydrogel framework (Radulescu et al., 2022).

*Liquid absorption specificity:* one of the important hydrogels’ characteristics is their ability to swell when the system is in contact with water or a thermodynamically compatible solvent. When the hydrogel structure contacts with solvent molecules, they enter the polymer network. Due to hydration, the polymer chains are weakened, and as a result, an entire system expands. This process is supported by existing osmotic forces, while the elastic force of hydrogel balances a polymer network and prevents its deformation, which leads to equilibrium (equilibrium water content) without additional swelling (Bashir et al., 2020; Zhang et al., 2023).

*Mechanical strength and stiffness:* The mechanical properties of hydrogel scaffold must match a specific tissue to enhance cell adhesion and precisely fill damaged tissue. The stiffness of the hydrogel matrix affects the stem cell differentiation. The necessary mechanical requirements for each hydrogel are specific to a target tissue type (Khaing et al., 2016; Foster et al., 2017).

*Biodegradability:* biodegradability is another important property for hydrogel development as they require controlled degradation and resorption *in vivo*. Degradation of the hydrogel matrix is known as a chemical process, but it can also be driven by a dynamic physical stimulus that affects cell function and behaviour. The main purpose of this process is to provide the ability to mimic ECM and enhance tissue regeneration (Oliva et al., 2017). The biodegradability of hydrogels is manifested in the ability to break down in the recipient’s body, and is used to encapsulate medicinal substances for several days or weeks to extend their shelf life (Chen et al., 2019).

*Self-healing* is an unusual property of hydrogels, which manifests itself in the ability to restore its original structure after exposure to exogenous factors. This property of hydrogels is important for parenteral therapy. In addition, self-healing of hydrogels increases their period of existence in the body and increases the effectiveness of any therapy (Chyzy and Plonska-Brzezinska, 2020).

*Reduction of stress* by ionic and covalent hydrogels occurs by splitting and reformatting crosslinks within the polymer and water migration. This property of hydrogels promotes SC growth, their proliferation and differentiation into different types of cells (Li et al., 2018).

Most authors in their research even indicate some functional features of an ideal hydrogel:

➢ Colorless, odourless, absolutely non-toxic (Marin et al., 2013);
➢ The highest capacity for biological degradation without toxic substances formation after degradation. For example, PLGA hydrogel, which was approved by the FDA, however, its degradation products (obtained *in vivo*) turned out to be toxic, which significantly limits its clinical use (Oliva et al., 2017);
➢ pH neutrality after swelling in water (Li et al., 2018);
➢ The highest strength and stability in a swelling environment and during storage (Bashir et al., 2020);
➢ Ability (if necessary) to rewet: hydrogel must be able to dehydrate the solution or retain it; depending on application requirements (Ahmed, 2015; Yang et al., 2022);
➢ The greatest capacity for hydration (maximum equilibrium swelling) in physiological solution (Bashir et al., 2020);
➢ The highest absorbency under load (AUL) (Chai et al., 2017);
➢ Desired absorption rate (desired particle size and porosity) depending on application requirements (Nicodemus and Bryant, 2008; Slaughter et al., 2009);
➢ The lowest soluble content and residual monomer (Bashir et al., 2020);
➢ Photostability (Bashir et al., 2020; Yang et al., 2022).

## 4 Advances of hydrogel combined with neural stem cells in SCI

Stem cell transplantation has always been considered effective, however, it can cause an active immune response and cannot ensure the presence of SC at the injury site for a long time, which directly affects the therapeutic effect (Perale et al., 2011). Therefore, a combination of stem cells and polymer matrices is currently considered the most optimal approach for injured nerve tissue regeneration, in particular, SC (Yang et al., 2020; Lin et al., 2021; Cheng et al., 2022; Li et al., 2022).

Every day there are more and more *in vitro* and *in vivo* studies devoted to combining hydrogels with NSCs (encapsulation of SCs) (Li et al., 2012; Li et al., 2022 J.; Garrudo et al., 2021; Yao et al., 2021; Yuan et al., 2021; Afsartala et al., 2022; Chen et al., 2022; Albashari et al., 2023; Ji et al., 2023; Jia et al., 2023; Liu et al., 2023; Yang et al., 2023). Especially NSCs which are a unique and effective tool for nerve tissue regeneration. This type of stem cell has three main characteristics: an ability to self-renew, neural tripotency—an ability to differentiate into the main lineages of neural cells, i.e., astrocytes, oligodendrocytes, and neurons, as well as an ability to regenerate *in vivo* (Casarosa et al., 2014; Assunção-Silva et al., 2015).

It has been proven that NSCs in injured SC differentiate into astrocytes and oligodendrocytes, which promotes remyelination. Neurons, into which NSCs differentiate, migrate rostrally and caudally at the contusion site of rat SC, which contributes to the improvement of locomotor functions. NSCs also contribute to the strengthening of recipient neuronal axons (Assunção-Silva et al., 2015). In addition to the fact that NSCs (encapsulated) within the matrices in injured SC differentiate into neurons and oligodendrocytes, they are also able to migrate in the injury site, enhance neuronal regeneration and reduce inflammatory processes in SC (Skop et al., 2014; Kandilogiannakis et al., 2021).

In the recipient, NSCs promote the recovery of damaged areas of nervous tissue by providing nearby cells with trophic substances, activating endogenous regeneration, or by directly replacing neurons and glial cells. Biodegradable polymers, in turn, serve as a framework for the axonal network restoration and at the same time act as a reservoir for progenitor cells. It was found that both natural and synthetic biomaterials contribute to NSCs’ survival, as they serve for the latter as a microenvironment with controlled properties for growth and differentiation (Kim and Jung, 2016; Zou et al., 2020).

It is known that NSCs growing in the PLGA matrix were used to restore axonal networks for lateral incision injury in rats. Already 70 days after implantation, experimental animals showed higher physical activity, and histological evaluation showed a decrease in the glial scar size. Moreover, studies using NPC-PLGA constructs, which were implanted into the fully dissected rat SC, showed the formation of a greater number of axons inside the polymer, in contrast to implants devoid of stem cells. Chitosan-NPS-constructs on the 7th day after implantation in a completely severed rat SC contributed to the restoration of nervous tissue structural integrity due to the high survival of stem cells and their active differentiation into astrocytes and oligodendrocytes (Shrestha et al., 2014). However, despite the efficiency of use for the restoration of damaged nerve tissue, the above-mentioned combined systems are characterized by a significant drawback—low survival and the impossibility of controlled differentiation of NSCs. Therefore, to improve NSCs’ state in the matrix: their survival, engraftment and integration into the recipient`s tissue, hydrogels are now used. As research has shown, one of the most common hydrogels is fibrin hydrogels, which can mimic natural environmental conditions for NSCs, which further differentiate into neurons, astrocytes, and oligodendrocytes (Marquardt and Heilshorn, 2017).

The use of hydrogels in combination with stem cells depends on the mechanical and biological properties of matrices. Thus, biochemical signals and interactions between stem cells and the ECM directly affect the SC’s shape: stiffness of the extracellular environment directly affects the cells (Chamkouri, 2021). Stem cells, which are cultivated together with hydrogels of a rigid structure, are characterized by a more developed cytoskeleton, proliferate faster and migrate within the matrix. On the other hand, non-toxicity, high viscosity, ability not to cause an immune response, stability and biodegradability of hydrogels contribute to higher adhesiveness, proliferation and growth of stem cells (Chamkouri, 2021).

In general, the compatibility of stem cells with hydrogels is influenced by such physical characteristics of matrices as stiffness, porosity, elasticity, architecture, adhesiveness, and biodegradability. *The stiffness* of hydrogels has a positive effect on stem cell morphology, proliferation and differentiation. Stem cells respond to the degree of matrix stiffness by triggering FAK, 170–172 Rho/ROCK, 173,174 YAP/TAZ, 26,175 Wnt/β-catenin signalling cascades, which cause their further differentiation (Cao et al., 2021). *The porosity* of hydrogels also affects stem cells’ properties, namely, their cytoskeleton reorganization, distribution of organelles in cells, chromatin and nuclear membrane proteins reorganization, and this in turn affects stem cells morphology, their migration, differentiation and germination in the recipient tissue (Li et al., 2012; Cao et al., 2021). *The elasticity* of hydrogels also has a positive effect on stem cell sprouting, proliferation and differentiation (Cao et al., 2021). *The architecture* (diameter and arrangement of fibrils) determines how stem cells will contact each other (Cao et al., 2021). *Biodegradation*: in contrast to non-biodegradable hydrogels, biodegradable matrices contribute to better contact of stem cells and their germination (Teng et al., 2002; Cao et al., 2021). *Stem cell adhesion sites* (formed by α2β1 integrins, CD44 proteoglycans, and receptor tyrosine kinase) by integrins in the matrix also affect stem cell survival by inducing (Straley et al., 2010; Cao et al., 2021).

It is known that hydrogels of both natural and synthetic origin are used for stem cell encapsulation. These can be collagen, fibrin, keratin, agarose and alginate (alginate-gelatin) hydrogels, chitosan and chondroitin sulfate matrices, hydrogels based on desired rubber and based on hyaluronic acid, Polyethylene Glycol (PEG) and Poly (N-isopropylacrylamide) (PNIPAAm) (Kandilogiannakis et al., 2021; Wang Y. et al., 2022).

As research results show, stem cell encapsulation provides a semi-permeable barrier for cells, which allows the absorption of nutrients and protection from harmful factors (Costa et al., 2022). An important advantage of stem cell encapsulation to prevent immune response occurrence is stem cell “masking” from recipients’ antibodies due to the semi-permeability of the matrix itself. Thereafter, such immunomodulation prolongs stem cell lifespan (Hasturk and Kaplan, 2019). Hydrogels also improve stem cells’ survival and proliferative capacity during long-term cultivation due to the stimulation of their natural resources (Bajaj et al., 2014). The creation of a semipermeable environment for stem cells by hydrogels also creates conditions for growth and activation of differentiation due to the supplying of appropriate exogenous factors. Such encapsulation also prolongs the duration of stem cells and medicinal substance’s therapeutic effect. Physical protection of stem cells against mechanical damage based on structural elements of hydrogels and crosslinks between them is considered another significant advantage of stem cell encapsulation, which determines this ability (Kandilogiannakis et al., 2021). Stem cell encapsulation is also effective for the cryoprotection of stem cells within cryogels (Bajaj et al., 2014; Hasturk and Kaplan, 2019; Kandilogiannakis et al., 2021; Costa et al., 2022). Therefore, well-spent that many authors claim that stem cells are always in a stable state within the hydrogel, and the hydrogel creates optimal conditions for the ECM of SC tissue (Bajaj et al., 2014; Pérez-Luna and González-Reynoso, 2018; Hasturk and Kaplan, 2019; Kandilogiannakis et al., 2021; Khayambashi et al., 2021; Lee et al., 2021; Costa et al., 2022; Huang et al., 2022).

It was established that NSCs/NPCs within hydrogels, in particular, alginate ones, in injured SC differentiate into neurons (40.7%), astrocytes (26.6%) and oligodendrocytes (28.4%) (Zhou et al., 2022). Such an indicator of differentiation leads to an increase in neuronal connections number and improves motor functions. iPSCs also differentiate into glial cells and neurons, so they are considered an alternative NSC type of stem cell, although their ability to multidirectional differentiation and transformation into cancer cells significantly limits the possibilities of their use. MSCs are often used to restore SC tissue due to their multidirectional differentiation, regulated immune response, and biological anti-inflammatory effects; an ability to replace damaged not only bone, cartilage and myocardium but also nerve tissue. The combination of MSCs with hydrogels promotes anti-inflammatory and antioxidant properties, which in turn activates endogenous NSCs’ survival and proliferation (Cai et al., 2023).

It has been shown that the use of hydrogels in combination with NSCs for SC regeneration contributes to their differentiation into neurons with subsequent involvement of the recipient’s lost neurons. Such NSCs within the hydrogel secrete neurotrophic growth factors, and cytokines, which contribute to inflammatory reaction reduction, damaged SC cells restoration, reduction of demyelination level and axon damage and, as a result, the subsequent restoration of the body’s motor functions (Lv et al., 2022).

Hydrogels in combination with embryonic stem cells (ESCs) are also used to enhance SC tissue regeneration and angiogenesis, enhance Tuj1, MAP2 and Syn expression, reduce immune response, and as a result—restore motor function. ESC-derived motoneurons within composite hydrogels are considered the most effective and least invasive method for regeneration (Lv et al., 2022). ESCs differentiate into neural and glial stem cells within hydrogels after implantation into the injured SC. In the injured SC of rats, ESCs migrate to the injury site and differentiate mainly into oligodendrocytes and astrocytes (Assunção-Silva et al., 2015).

iPSCs-derived neurospheres within hydrogels survive, migrate and differentiate into the main neural cell lines in injured SC. After transplantation, the expression of neurotrophic factors, activation of angiogenesis, restoration of axons, and remyelination at the injury site are noted. In the injured SC of nonhuman primates, iPSCs differentiate into neurons, astrocytes, and oligodendrocytes without degenerating into cancer cells (Assunção-Silva et al., 2015).

In another investigation, DNA hydrogels with NSCs were implanted in the SC (after 2-mm transection of rat SC); and already on the 8th week of observation, improvement of motor functions of both limbs was noted (Lv et al., 2022). And when HAMC hydrogel + rPDGF-A + NSCs were implanted into damaged SC, a decrease in SC damage area, and an increase of neurons and oligodendrocytes survival in intact areas of SC were established (Lv et al., 2022). During the implantation of collagen hydrogel + EGF + NSCs, a significant number of preserved nerve cells, reduction of glial scar size and, accordingly, its pressure on the adjacent tissue, were noted (Lv et al., 2022). It was also shown that hydrogels based on hyaluronic acid, compatible with embryonic NSCs, reduce SC damage area (Lv et al., 2022).

When GelMA/ECM hydrogel + NSCs were implanted in rat SC with complete transection, it was established that the GelMA/ECM matrix promotes axon sprouting as early as 8 weeks after implantation (Chen et al., 2022). And *in vitro* (cultivated for 14 days), such a matrix enhances NSC adhesion and differentiation. This type of matrix is synthesized from gelatin and methacrylic aldehyde, due to which its proteolytic biodegradability and cell attachment sites correspond to those of the natural ECM (Chen et al., 2022).

After complete transection of rat SC at T8-T10, for nerve tissue regeneration, AFG hydrogel (consisting of fibrinogen and poly (ethyloxide)) was also transplanted together with E14 NSCs of rats’ hippocampus. It was found that such a hydrogel, compatible with NSCs, reduces the level of inflammation of damaged tissue, promotes differentiation of NSCs into glial cells and neurons, and also promotes angiogenesis at the injury site (Yang et al., 2023).

NGP hydrogels (based on amino-gelatin) are also used together with NSCs to restore SC tissue after injury. Thus, E16 NSCs of rat neocortex were cultured together with NGP hydrogel for 7 days. Such constructs were implanted in the rat SC at T9-T10 level after excision (2-mm) trauma. It was shown that NGP promotes neurogenic differentiation of NSCs and inhibition of glial differentiation *in vitro*. The implantation of such structures contributes to axons sprouting, and their myelination strengthening, which in turn contributes to the improvement of hind limb locomotor functions (Liu et al., 2023). Similar results were obtained when pHPMA hydrogel with NSCs was implanted in rats SC after hemisection. Namely: an implant area was closely related to the recipient`s tissue; the recipient’s cell processes freely grew into the implant itself; NSCs populated in hydrogel differentiated into neurons; a dense network of neuron processes was visible inside the implant. Preliminary *in vitro* results showed that pHPMA hydrogel promotes NSC differentiation into neurons and inhibits their differentiation into glial cells, in particular, into astrocytes (Rybachuk et al., 2023a).

When implanting CSMA-hydrogels in combination with NSCs in rat SC after its contusion, it was shown that the combination of hydrogel with NSCs contributes to functional recovery of the SC cavity after injury and neurogenesis development with the prevention of fibroglial layer formation. And reduced level of NSC differentiation into astrocytes may be a consequence of the encapsulation of cells in the composition of a hydrogel (Liu et al., 2019).

It is known that NSCs in the composition of hydrogel matrices are also implanted in SC with chronic injury, both in experimental models and in clinical cases. In particular, it was shown that the presence of NSC affects the reduction of cyst size; and provides axon regeneration through a synergistic effect on fibroblasts or neuroepithelial stem cells through EGF, bFGF, PDGF-AA and NT-3 synthesis; NSCs can differentiate into all three types of glial cells to regenerate neuronal pathways, demyelinated axons, and can provide trophic support for endogenous cells (Suzuki et al., 2022). Thus, laminin-coated hydrogels with iPSCNPs (iPSC-derived neural progenitor cells) in chronic SCI in rats reduce the cavity area in the SC tissue. iPSCNPs and oligodendrocyte progenitor cells differentiate within the matrix and form long axons to communicate with each other. iPSCs in hyaluronic and gelatin hydrogels accelerate neurite elongation and induce spontaneous neuronal differentiation (Lv et al., 2022).

Undoubtedly, such combinations of hydrogel + NSC have prospects for their further clinical application. Namely, the improvement of axon sprouting, their remyelination and the formation of synapses contribute to the improvement of locomotor and sensory functions of the limbs of a patient with SCI (Zeng, 2023a). In the case of injury to the SC (for example, at the lower thoracic level) and implantation of the hydrogel + NSC complex, the appearance of elements of deep sensitivity in the proximal areas of the buttocks is possible, which will contribute to the further improvement of motor functions. Due to the presence of stem cells, there is a decrease in inflammatory processes in the area of the injury (Freyermuth-Trujillo et al., 2022), which also contributes to the acceleration of functional recovery. In general, a systemic improvement in the condition of patients after SCI is possible: improvement in the functioning of internal organs, absence/reduction of the need for catheterization, and partial or even complete independence.

First and foremost, the hydrogel implanted in the injured region of SC has a hemostatic effect, which helps to reduce both the primary and secondary inflammatory responses (Lv et al., 2022). Later, axons sprouting, reinnervation, and remyelination occur, as the hydrogel provides the necessary supportive mechanical microenvironment by connecting the edges of the damaged nerve tissue (Hejcl et al., 2008; Hong et al., 2017; Silva et al., 2021; Lv et al., 2022; Park et al., 2022). In parallel, NSCs (in particular, their secretome) also have a positive effect on all the above-mentioned processes by a paracrine mechanism; differentiate, in particular, into neurons that are actively integrated into the recipient’s neuronal networks (Hey et al., 2023). All of the above significantly improves the morphological structure of the damaged nerve tissue and therefore contributes to the recovery of motor function after SCI (Hong et al., 2017; Silva et al., 2021; Park et al., 2022).

### 4.1 Problems and possible limitations in the use of hydrogel with NSCs in SCI

Today, there is a significant variety of natural and artificial materials that have potential prospects for use in tissue engineering and neuroengineering in particular (Boni et al., 2018; Kim et al., 2023). Each material (matrix) has both general characteristics (physical, chemical, biochemical, etc.) and certain individual characteristics, which makes them as suitable for use as possible (Akhtar et al., 2023; Kim et al., 2023; Nasser et al., 2024). However, among all the positive aspects, limitations in their further use often arise, particularly in clinical application (Niyonambaza et al., 2019; Akhtar et al., 2023; Socci et al., 2023).

An important requirement for any matrix is an absence of immune reaction initiation, and, according to chemical and physical properties, the ability to support the recipient`s tissue (Boni et al., 2018; Bian et al., 2023). Therefore, one of the main characteristics is the biodegradation of such matrices (Meereboer et al., 2020; Socci et al., 2023). The formation of stable intermediate products should accompany biodegradation. Stability means the absence of the body’s immune reaction to them, the absence or slight toxicity and their easy transformation into substances that can be used by the body in synthesis processes or easily removed from it as needed (Subramanian et al., 2009; Socci et al., 2023).

As already mentioned above, matrices are of biodegradable and non-biodegradable types (Subramanian et al., 2009; Boni et al., 2018; Geevarghese et al., 2022). A feature of the first is the possibility of decomposition inside the recipient`s tissue, and this is their advantage because, in the process of tissue regeneration, no obstacles to the growth of cells are created (Geevarghese et al., 2022; Oleksy et al., 2023). However, they also have a significant drawback—an impossibility of regulating the toxicity of their decomposition products (Gu, 2021; Oleksy et al., 2023). Another problem in the production of matrices of this type is the heterogeneity of the material and the difficulty of its cleaning (Gu, 2021; Kirillova et al., 2021). Incomplete clearance of the material can lead to strong immune response development in already damaged tissue (Kasravi et al., 2023). Their development is more complicated and takes more time because it is necessary to achieve minimum toxicity of decay products and approximate synchronization of the decomposition time of the substance with the time of tissue regeneration. Such substances include chitosan, poly-α-hydroxy acids and poly-δ-butyrate (Straley et al., 2010).

Another type of matrix, that is, non-biodegradable, is characterized by mechanical strength and elasticity, but this strength can be a disadvantage in some cases, for example, sometimes such matrices can limit cell growth (Boni et al., 2018; Krishani et al., 2023). After all, non-biodegradable matrices are mainly made of synthetic materials (Phiri et al., 2023), and the constant presence of an implant made of such substances can increase the risk of inflammation, and can often lead to compression of the nerve tissue section, which in the long run will necessitate another operation to remove or replace such implant (Reddy et al., 2021; Flores-Rojas et al., 2023; Khodaei et al., 2023). For example, silicone is a biocompatible material, but it is not used *in vivo* due to its non-porous structure and frequent cases of its compression of the lesion site, which significantly impairs the recovery process (Bruckmann et al., 2022; Nuwayhid et al., 2024). Therefore, as an alternative to silicone, PVC is more often used, which is semipermeable and retains stability *in vivo*. However, despite its biocompatibility, PVC does not have a high degree of adhesiveness, which limits its use in the future (Chen et al., 2021).

pHEMA, a matrix of synthetic origin, is known not to biodegrade. In addition, the presence of calcification and a prolonged inflammatory reaction after implantation of such a matrix lead to a slowdown in the regeneration of axons (Ma X. et al., 2022).

Although, as most authors point out, polymer matrices still have several advantages for SC regeneration. Thus, matrices repeat the natural structure of the extracellular matrix and stimulate the growth of axons, promoting axon growth. In addition, matrices can serve as a basis for the transplantation of stem cells of various types, growth factors and medicinal substances (Cheng et al., 2022; Li W. et al., 2022). For strengthening the regenerative properties of matrices, researchers are still working on the optimization of mechanical properties, increasing the adhesiveness of cells, ability to biodegradation and electrical activity (Straley et al., 2010). Hydrogel matrices based on natural components, in particular, polysaccharides (collagen, gelatin, alginate, agarose, etc.) deserve special attention. Such matrices have some advantages: susceptibility to biodegradation, hydrophilicity, and porosity of the structure, which allows for imitation of the natural microenvironment of nervous tissue (Wang Y. et al., 2022).

On the other hand, stem cell transplantation has always been considered effective (Zeng, 2023b; 2023a), however, sometimes it causes an active immune response and cannot ensure the presence of stem cells at the injury site for a long time, which directly affects the therapeutic effect (Shang et al., 2022; Zeng, 2023a). Therefore, nowadays hydrogels are most often used in combination with different types of stem cells: cells are always in a stable state within the hydrogel, and the matrix creates optimal conditions for the extracellular matrix of SC tissue (Khayambashi et al., 2021; Peng et al., 2023). However, data indicate that stem cells populated in the matrix cannot stay in it permanently, despite the volume and tunnelling of the matrix itself (Lv et al., 2022).

Regarding NSCs, the biggest drawback in their use is bioethical norms that do not allow the use of NSCs in the clinic, it is impossible to use them as autologous material, ability of this type of stem cells to transform into cancer cells has also been confirmed (Pereira et al., 2019). Also, a limitation is that it is difficult to obtain enough NSCs to regenerate damaged nervous cells, as endogenous NSCs reside in a restricted niche of the CNS and have a limited ability to proliferate *in vivo* compared to other stem cells (Behnan et al., 2017; Kim et al., 2022). Although it was shown that NSCs within hydrogels differentiate into astrocytes, oligodendrocytes and neurons, which increases the number of neuronal connections and improves motor functions (Cai et al., 2023).

An alternative to NSCs is iPSCs, which also differentiate into glial cells and neurons (Cai et al., 2023). After implantation in the area of SC damage, iPSCs-derived neurospheres within hydrogels survive, migrate and differentiate into neurons and glial cells (Kong et al., 2021; Xue et al., 2021). After such implantation, the expression of neurotrophic factors, activation of angiogenesis, restoration of axons, and remyelination at the injury site are noted (Assunção-Silva et al., 2015). It has been proven that in the injured SC of non-human primates, iPSCs differentiate into neurons, astrocytes, and oligodendrocytes without degenerating into cancer cells (Assunção-Silva et al., 2015).

MSCs are often used for the restoration of SC tissue due to their multidirectional differentiation, regulated immune response and biological anti-inflammatory effects, therefore they can replace damaged not only bone, cartilage tissue and myocardium, but also nerve tissue. The combination of MSCs with hydrogels promotes anti-inflammatory and antioxidant properties, which in turn activates the survival and proliferation of endogenous NSCs (Perale et al., 2011; Cai et al., 2023).

However, despite the indicated problems and limitations in the use of matrices, hydrogels, of natural and synthetic origin, and NSCs, the data obtained made it possible to more thoroughly investigate the mechanisms of cellular interactions at the site of tissue damage (Yang et al., 2022; Cai et al., 2022; Cai et al., 2023) and accelerated experimental and, to some extent, clinical progress in the field of neuroengineering.

## 5 Conclusion

In recent years, more and more data have been accumulated that indicate the effectiveness of complex approaches for SC nerve tissue regeneration usage. Such a complex approach lies in the application of hydrogels populated with stem cells, in particular NSCs. However, there are still a lot of questions to be clarified for the possibility of further application of this approach in clinical practice (for hydrogel: choose the most suitable/ideal sample in terms of nervous tissue characteristics; for stem cells, first of all, establish an amount of population per unit volume of hydrogel to achieve the maximum positive effect). However, the prospect of such a complex method of SC nerve tissue restoration is undeniable.
